# Fat matters: paraspinal muscle quality is associated with electromyographic fatigability and endurance in lumbar spinal stenosis

**DOI:** 10.1016/j.xnsj.2026.100916

**Published:** 2026-06-11

**Authors:** David Koch, Corina Nüesch, Gerold Müller, Peter Stauffert, Annegret Mündermann, Alice Caimi, Dominika Ignasiak, Stefan Schären, Stephen J Ferguson, Hans-Joachim Wilke, Cordula Netzer

**Affiliations:** a Department of Spine Surgery, University Hospital Basel, Spitalstrasse 21, Basel 4031, Switzerland; b Department of Orthopedics and Traumatology, University Hospital Basel, Spitalstrasse 21, Basel 4031, Switzerland; c Department of Biomedical Engineering, University of Basel, Hegenheimermattweg 167b, Allschwil 4123, Switzerland; d Institute of Orthopedic Research and Biomechanics, Centre for Trauma Research, Ulm University Medical Centre, Helmholtzstrasse 14, Ulm 89081, Germany; e Department of Clinical Research, University of Basel, Spitalstrasse 8, Basel 4031, Switzerland; f Department of Teaching, Research and Development, Schulthess Clinic, Lengghalde 2, Zurich 8008, Switzerland; g Institute for Biomechanics, ETH Zurich, GLC, Gloriastrasse 37/39, Zurich 8092, Switzerland

**Keywords:** Lumbar spinal stenosis, Paraspinal muscles, Fatty infiltration, Electromyographic fatigability, Electromyography, Median frequency, Biering-Sørensen test, Magnetic resonance imaging, Fat fraction, Multilevel modeling

## Abstract

**Background:**

Paraspinal muscle degeneration with fatty infiltration and atrophy is well-documented in symptomatic lumbar spinal stenosis (sLSS). However, it remains unclear whether these structural alterations are associated with paraspinal muscle function. This study investigated associations between paraspinal muscle morphology, surface electromyography (sEMG)-derived fatigability and endurance capacity in sLSS.

**Methods:**

A total of 105 preoperative patients with sLSS underwent quantitative lumbar MRI (VIBE Dixon sequence) and a modified Biering-Sorensen test with concurrent sEMG. Fat fraction, total and lean cross-sectional area (CSA) were derived from machine-learning-assisted segmentation at L1, L3, and L5. Normalized median frequency (MDF) slope, MDF decline, and initial MDF were derived from sEMG. Spearman correlations and multilevel linear models adjusted for age, sex, BMI, and endurance, examined associations between morphology and fatigability.

**Results:**

Mean endurance was 101.2 ± 72.5 seconds. Fat fraction was consistently high in the multifidus (46%–49%) and increased caudally in the erector spinae (31% at L1 to 61% at L5), correlating negatively with endurance bilaterally (−0.34 ≤ρ≤ −0.50). In multilevel models, fat fraction was independently associated with more negative MDF slopes (−0.005, 95% CI [−0.011; −0.001]), greater MDF decline (0.619 [0.15; 1.12]), and greater initial MDF (9.883 [9.01; 10.65]). Total CSA was associated only with initial MDF.

**Conclusions:**

Paraspinal fat fraction was associated with the rate and magnitude of MDF decline, independent of muscle size, endurance, age, and sex. These findings suggest that morphological changes in paraspinal muscles have functional correlates that may contribute to activity limitations in sLSS. Muscle quality appears more relevant than muscle quantity for fatigability in this population.

## HIGHLIGHTS

• Paraspinal muscle fat fraction predicted electromyographic fatigability.
• Fat fraction predicted fatigability independent of age, sex, BMI and endurance.
• Greater fat fraction was associated with lower trunk extension endurance.
• Quantitative imaging and bilateral EMG enabled level- and side-specific assessment.
• Muscle quality mattered more than quantity for electromyographic fatigability.

## Introduction

Symptomatic lumbar spinal stenosis (sLSS) is a degenerative spinal disorder that involves constriction of neural pathways within the central spinal canal, the lateral recesses, or intervertebral foramina, often resulting in nerve root compression [1]. The clinical presentation of sLSS extends beyond localized back pain, often manifesting as neurogenic claudication: leg pain, heaviness, and weakness that worsen with walking [2] and lumbar extension [3]. To alleviate discomfort, patients commonly adopt forward-flexed postures and avoid lumbar extension [3,4]. Over time, these postural adaptations and reductions in physical activity may affect the paraspinal musculature. While disability in sLSS has traditionally been attributed to neural compression, emerging evidence suggests that alterations in paraspinal muscle structure represent an additional component of the condition [5].

Studies involving magnetic resonance imaging (MRI) provide consistent evidence of paraspinal muscle degeneration in patients with sLSS. Compared to age-matched controls, patients with sLSS demonstrate atrophy of the multifidus and erector spinae muscles, with cross-sectional area (CSA) reductions frequently exceeding 20% to 30% [5,6]. Fatty infiltration of the paraspinal muscles is also notably greater in this population [5,[7], [8], [9]]. However, similar to the well-established mismatch between stenosis severity and symptom intensity in sLSS [10], paraspinal muscle morphology is not robustly associated with pain severity. A comprehensive systematic review [11] reported moderate-to-high-certainty evidence of no consistent link between paraspinal muscle morphology and back or leg pain across multiple muscle groups, with greater multifidus fatty infiltration most consistently associated with the presence of LSS rather than with symptom intensity [11].

This apparent mismatch between muscle morphology and pain raises important questions regarding the clinical relevance of paraspinal muscle degeneration in sLSS. Instead of contributing directly to pain, structural changes in the paraspinal muscles may be associated with altered electromyographic fatigability and functional endurance during sustained trunk extension. These mechanisms may be particularly relevant in sLSS, where walking intolerance, postural demands, and activity-dependent symptoms dominate the clinical presentation. Despite robust evidence of morphological compromise, the functional implications of these changes remain poorly understood [5,11].

Functional assessment of the paraspinal muscles provides insight into their capacity to contribute to spinal stability during daily tasks. The Biering-Sørensen test is a validated measure of paraspinal muscle endurance and has demonstrated strong associations with disability and recurrent low back pain [12]. Surface electromyography (sEMG) during sustained contractions offers complementary information on electromyographic fatigability. While sEMG does not directly measure force output, changes in the frequency content of the signal—typically quantified by the decline in median frequency (MDF) [[13], [14], [15]]—reflect underlying physiological processes associated with fatigue, including slowing of muscle fiber conduction velocity [[16], [17], [18]]. In this paper, electromyographic fatigability refers specifically to these sEMG signal-based manifestations associated with fatigue processes, rather than a direct measure of force output decline. Although paraspinal endurance and fatigability have been studied in chronic low back pain populations [19], their relationship to the well-documented paraspinal muscle morphological changes observed in sLSS remains unclear.

The purpose of this study was to investigate pre-operative associations between paraspinal electromyographic fatigability parameters, paraspinal muscle endurance, and paraspinal muscle morphology in patients with sLSS.

## Material and methods

This institutional review board-approved cross-sectional analysis was conducted as part of a larger umbrella study [20] on functional biomechanics and clinical symptoms in patients with sLSS (ethics approval: Ethics Committee Northwest and Central Switzerland, EKNZ 2022-01170; clinicaltrials.gov NCT05523388). All participants provided written informed consent.

### Participants

We recruited 122 patients with sLSS scheduled to undergo decompression surgery, of whom 105 completed the study protocol. Patients were identified through the institutional surgery schedule. Eligibility was determined by the study physician based on predefined inclusion and exclusion criteria. Inclusion criteria were age greater than 30 years, body mass index (BMI) below 35 kg/m^2^, a confirmed radiological diagnosis of sLSS, symptom duration of at least 6 months, presence of intermittent neurogenic claudication with limitation of walking ability due to symptoms in the lower back and/or in one or both legs and failure of conservative treatment. Exclusion criteria were inability to provide informed consent, prior spine surgery, reliance on walking aids, other neurologic disorders affecting gait, and pregnancy.

### Study procedures

First, patients digitally completed the Oswestry Disability Index (ODI) [21], Swiss Spinal Stenosis measure (SSS) [22,23] and Core Outcome Measures Index for the back (COMI) [24,25] using REDCap [26,27]. Subsequently, lumbar spine MR images were obtained with patients in the supine position using a 0.55 T MAGNETOM Free.max MRI scanner (Siemens Healthcare, Erlangen, Germany). The MRI protocol comprised standard clinical sequences, including sagittal T1- and T2-weighted turbo spin echo (TSE), transverse T2-weighted TSE, coronal T2-weighted STIR, and a transverse VIBE 2-point Dixon sequence. While VIBE Dixon slices were acquired horizontally without angulation, T2 sequences were acquired strictly axially to the curvature of the spine. Severity of central canal stenosis (Schizas classification, grades A-D [28]) and foraminal stenosis (Lee classification, grades 0–3 [29]) were rated at each level by three physicians, including one radiologist.

**Fig. 1:**
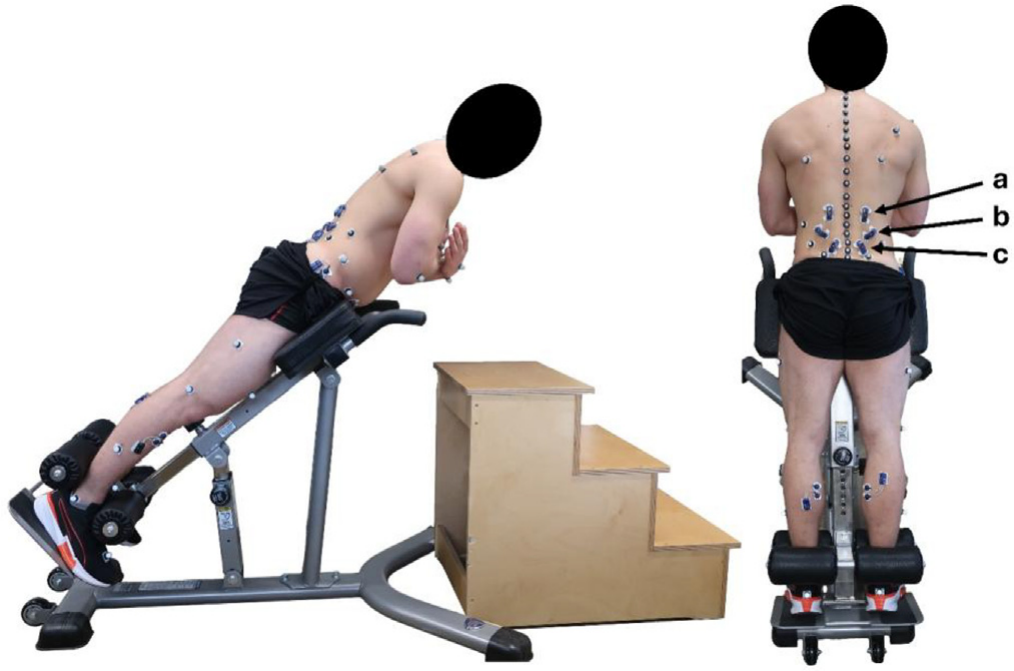
Modified Biering-Sørensen test setup (left) and placement of electromyography sensors (right). a: erector spinae (longissimus); b: erector spinae (iliocostalis); c: erector spinae (multifidus).

After MRI, patients underwent motion capture analysis in the hospital’s clinical motion laboratory. This included the patients performing a modified Biering-Sørensen test with concurrent sEMG recordings to assess paraspinal muscle endurance and paraspinal muscle fatigability. sEMG was recorded bilaterally using a wireless system (myon AG, Schwarzenburg, Switzerland; sampling rate 2400 Hz) from the erector spinae longissimus, iliocostalis, and multifidus muscles. Prior to electrode placement, the skin was shaved and cleaned with alcohol to minimize impedance, and electrode positioning followed the recommendations of the SENIAM project [30]. Owing to the high physical demands of the original horizontal Biering-Sørensen position, participants performed the modified test on a Roman chair inclined at 40°, corresponding to approximately 75% of the torque demand of the standard test [31] (Fig. 1). The procedure was conducted within individual tolerable pain limits and terminated if participants used their arms for support or deviated from the prescribed posture more than three times.

### Outcome measures

#### Electromyographic fatigability of paraspinal muscles

Test start and end were identified from motion capture marker data (Nexus 2.13, Vicon, Oxford, UK), and sEMG recordings were time-synchronized and automatically cropped to this interval. Signal postprocessing was performed in MATLAB™ (R2022b, MathWorks, Natick, MA, USA). Raw sEMG signals were band-pass filtered between 20 and 450 Hz using a fourth-order Butterworth filter [32,33]. For each muscle and side, the filtered signals were segmented into consecutive 1-second windows with 50% overlap [34,35]. Each window was transformed into the frequency domain using a Fast Fourier Transform, and the power spectral density was estimated via the periodogram. MDF was defined as the frequency that divides the power spectrum into two regions of equal power.

**Fig. 2:**
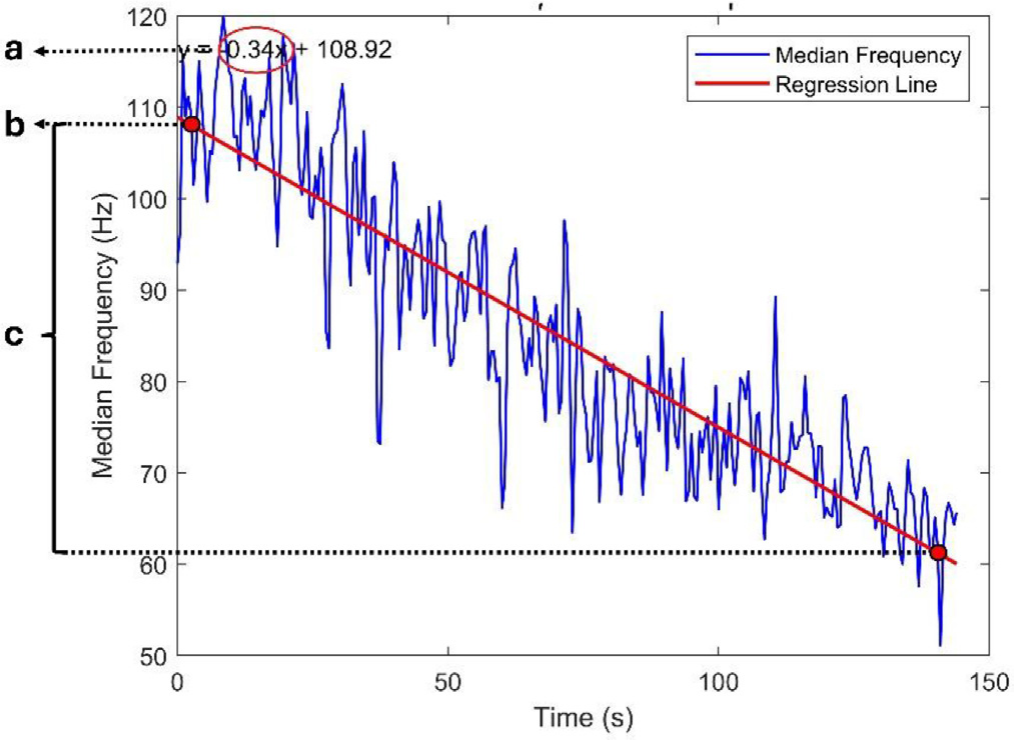
Fatigability metrics obtained from median frequency assessed during the duration of the modified Biering-Sørensen test. a: Median frequency slope (regression slope); b: Initial median frequency; c: Absolute median frequency decline.

MDF values were calculated for all analysis windows, resulting in continuous MDF time series that reflected the temporal evolution of the EMG signal throughout the test. Artefacts (typically isolated spikes from transient signal loss) were automatically identified and excluded, preserving the overall time series length. Paraspinal muscle fatigability was characterized using the following parameters, calculated separately for each muscle and side (Fig. 2):

a. **Normalized MDF slope [%/s]**—MDF slope was calculated by fitting a least-squares linear regression to the MDF time series, with the regression coefficient representing the rate of MDF change over time (Hz/s). To account for inter-individual differences in baseline frequency content, the slope was normalized by dividing it by the intercept of the regression line and multiplying by 100. This provided the relative rate of MDF change in percent per second (%/s) [36].
b. **Initial MDF [Hz]**—mean MDF across the first five valid analysis windows.
c. **Normalized MDF decline [%]**—calculated as the difference between the initial MDF and the mean MDF of the last five valid analysis windows, expressed as a percentage of the initial MDF to reflect the relative frequency decline during the test.

To ensure reliable estimation of sEMG fatigability parameters, normalized MDF slope and normalized MDF decline were excluded for participants with endurance times below 15 seconds, because these short test durations were associated with unstable frequency estimates and compromised parameter accuracy. Initial MDF values were retained for all participants, as these were not affected by test duration. This quality control criterion resulted in the exclusion of fatigability parameters from 11 patients.

#### Paraspinal muscle endurance

Paraspinal muscle endurance was defined as the duration in seconds that patients were able to maintain the correct test position during the modified Biering-Sørensen test.

#### Paraspinal muscle morphology

Paraspinal muscle morphology was quantitatively assessed using a machine-learning algorithm [37], iteratively retrained on manually corrected VIBE Dixon images (ITK-Snap [38], Version 4.2.0). Segmentation masks (T12 to S1) were generated for each patient and manually verified. The first 50 patients were segmented by one experienced rater, with subsequent corrections by two additional raters reviewed by the primary rater.

**Fig. 3:**
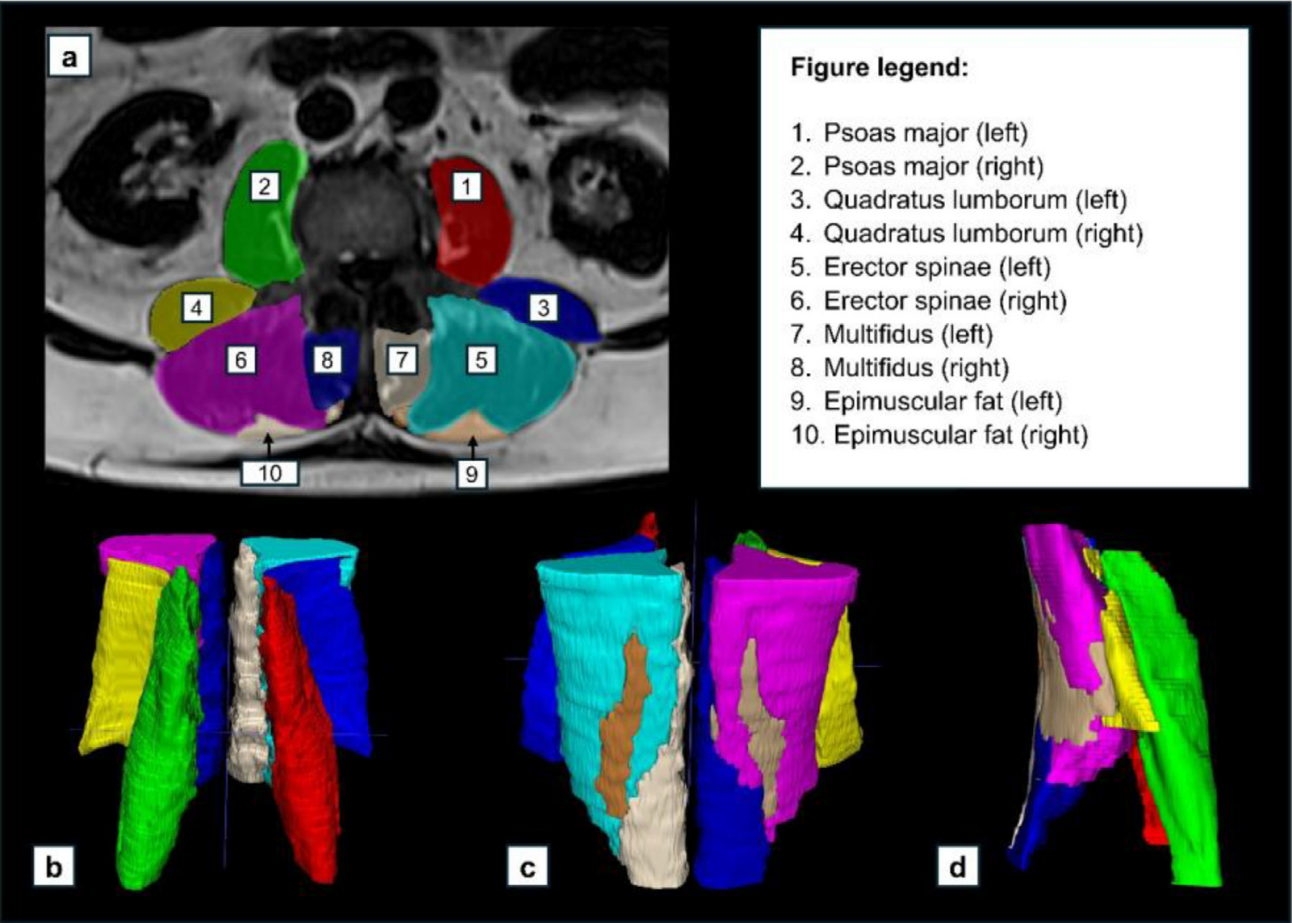
Illustration of MRI segmentation for assessing cross-sectional area, fat fraction and lean area. a) axial view. b) 3D view from anterior. c) 3D view from posterior. d) right lateral view.

Left and right erector spinae, multifidus, quadratus lumborum, and psoas major were separately segmented based on established guidelines [39,40] (Fig. 3). After finishing the segmentation masks, each MRI-slice was manually assigned to the appropriate vertebral body (L1-S1) or intervertebral disc (L1/L2 - L5/S1). The final segmentation masks were used to extract morphological parameters, including total muscle CSA, lean muscle CSA and fat fraction, derived from the fat and water images produced by the Dixon-based acquisition [[41], [42], [43]].

Following completion of MRI segmentation, three muscle morphology parameters were calculated for each slice:

1. **Total muscle CSA [cm2]**: Defined as the total CSA of the segmented muscle in the Dixon fat image, including both muscle tissue and fat. Calculated by multiplying the number of pixels within the segmentation mask by the in-plane pixel area.
2. **Lean muscle CSA [cm2]**: Muscle area free of fat infiltration, derived by subtracting the fat CSA from the total muscle CSA.
3. **Fat fraction [%]**: Quantifies fat infiltration within the segmentation mask and was derived from the chemical-shift-encoded VIBE Dixon images using the fat-only and water-only pixel intensities as $Fat\;Fraction\;\%=\frac{Fat\;pixel\;intensity}{Fat\;pixel\;intensity\;+\;Water\;pixel\;intensity\;}x100$

Per-level values were obtained by averaging across half of the slices corresponding to the superior intervertebral disc, all slices of the vertebral body, and half of the slices corresponding to the inferior intervertebral disc.

### Statistical analysis

Statistical analyses were performed in R (version 4.5.1, R Foundation for Statistical Computing, Vienna, Austria). Categorical variables are presented as counts and percentages (n, %), and continuous variables are summarized using mean, standard deviation (SD), minimum and maximum.

To ensure anatomical validity when relating sEMG-derived fatigability metrics to MRI-derived muscle morphology, each sEMG sensor was matched to the muscles it primarily recorded at the corresponding lumbar level. Specifically, the signal from the longissimus sensor placed at L1 was matched to L1 erector spinae and multifidus morphology; the iliocostalis sensor at L3 was matched to L3 erector spinae, quadratus lumborum, and multifidus morphology; and the multifidus sensor at L5 was matched to L5 erector spinae and multifidus morphology. The psoas major was excluded, as it does not contribute to trunk extension in the Biering-Sørensen test and no corresponding sEMG signal was recorded.

Associations between sEMG fatigability metrics and MRI-derived muscle morphology were examined using Spearman's rank correlation coefficient (ρ), as not all variables were normally distributed based on quantile-quantile plots and Shapiro-Wilk tests. Correlations were performed separately for the left and right sides. Correlations between the four sEMG-derived parameters (initial MDF, normalized MDF decline, normalized MDF slope, and endurance time) and the three MRI-derived parameters (total muscle CSA, lean muscle CSA, and fat fraction) were computed for each anatomically matched muscle. False discovery rate (FDR) correction was applied using the Benjamini-Hochberg procedure separately within each sensor-side combination. Statistical significance was defined as p < .05 after FDR correction.

Because each participant contributed multiple sEMG recordings across lumbar levels and sides, observations were not independent. To account for this hierarchical data structure, multilevel linear models were used with a two-level random-intercept structure: (1 | participant ID / side). Given that sLSS may affect paraspinal musculature asymmetrically, this structure captures both between-participant variability and systematic within-participant left-right differences.

Normalized MDF slope, normalized MDF decline, and initial MDF were analyzed in three separate models with identical predictors and random-effects structures. Primary predictors were fat fraction (%) and total muscle CSA (cm²) of muscles anatomically corresponding to the sEMG electrode. Lean muscle CSA was excluded as a predictor because it is mathematically determined by total muscle CSA and fat fraction; including all three would introduce multicollinearity. All models were adjusted for age, sex, BMI, and endurance time. Continuous predictors were z-standardized to facilitate effect size comparison.

Models were fitted using restricted maximum likelihood (REML) with the *lme4* package [44]. Ninety-five percent confidence intervals (CIs) were obtained via subject-level bootstrap resampling (1,000 iterations) using bias-corrected and accelerated (BCa) adjustment. Effects were considered statistically significant when the 95% CI excluded zero. Model assumptions were evaluated using simulation-based residual diagnostics (DHARMa package). Influential observations were identified using Cook's distance but none were excluded, as fatigability metrics lack clear physiological bounds and influential cases were primarily associated with short endurance times rather than implausible values.

## Results

### Cohort characteristics

Of the 105 patients who completed the protocol (Table 1), initial MDF values were available for all patients and sEMG fatigability parameters were available for 94 patients (11 excluded due to endurance times below 15 seconds).

**Table 1:** Cohort characteristics.

Cohort characteristics				
	Mean (SD)		Min.	Max.
Demographics				
Age (years)	70.1 (9.6)		36.8	85.1
BMI (kg/m²)	26.6 (4.3)		15.6	35.8
Sex—female (n (%))	54 (51.4%)			

Patient-reported Outcomes				
Core Outcome Back Index Score	6.0 (1.7)		2.6	9.5
Oswestry Disability Index	29.2 (12.7)		4	60
Swiss Spinal Stenosis questionnaire: Function subscore	9.9 (2.8)		5	16
Swiss Spinal Stenosis questionnaire: Symptoms subscore	20.4 (3.9)		12	31

Endurance time (seconds)	101.2 (72.5)		4.3	482.0

Stenosis severity cross-tabulation	Central Canal Stenosis (Schizas)			

Foraminal stenosis (Lee)	A	B	C	D

0 (None)	3*	1	1	3
1 (Mild)	3	6	9	9
2 (Moderate)	0	1	8	3
3 (Severe)	13	7	19	19

⁎ MRI-confirmed recessus stenosis. SD, standard deviation; Min:, minimum; Max, maximum; BMI, body mass index.

### Descriptive data

**Fig. 4:**
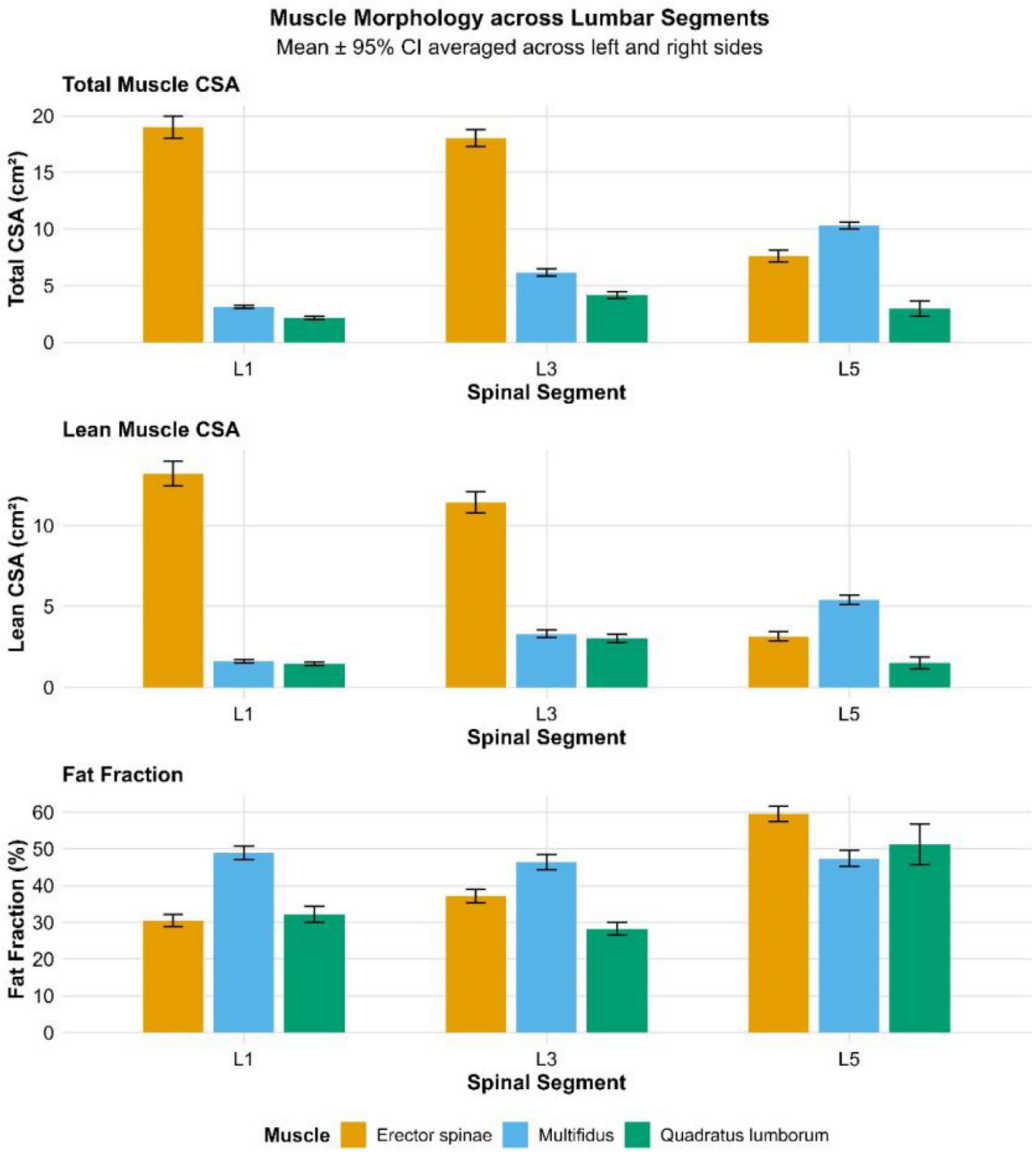
Variation in paraspinal muscle morphology across lumbar segments L1, L3, and L5. Data represent mean ± 95% confidence interval averaged bilaterally (n = 105). CI: Confidence interval; CSA: cross-sectional area.

Muscle morphology and fatigability parameters are summarized in Table 2 and Fig. 4. Fat fraction was consistently high in the multifidus (46%–49% across levels) and increased caudally in the erector spinae (31% at L1 to 61% at L5) and quadratus lumborum (28% at L3 to 55% at L5). Lean CSA of the erector spinae declined from L3 to L5, while total CSA was relatively consistent across most muscles and levels. sEMG-derived fatigability parameters varied considerably across patients. Normalized MDF declines ranged from 12 to 15% and were greatest for multifidus and longissimus. Initial MDF values were highest in the multifidus (∼94 Hz) and lowest in the iliocostalis (∼63 Hz). Normalized MDF slopes ranged from −0.13% to −0.17%/s across muscles.

**Table 2:** Descriptive statistics of muscle morphology and electromyographic fatigability parameters

		Left side			Right side		
MRI parameters*		Mean (SD)	Min	Max	Mean (SD)	Min	Max
**L1**							
Erector spinae	Total CSA (cm²)	18.96 (4.98)	10.63	34.66	19.07 (5.13)	10.50	37.64
	Lean CSA (cm²)	13.16 (3.92)	5.29	24.89	13.27 (4.04)	5.38	25.24
	Fat Fraction (%)	30.7 (8.3)	15.3	62.7	30.4 (9.1)	13.7	68.9
Quadratus lumborum	Total CSA (cm²)	2.11 (0.79)	0.32	4.17	2.18 (0.87)	0.01	4.10
	Lean CSA (cm²)	1.42 (0.61)	0.15	3.01	1.50 (0.64)	0.01	3.30
	Fat Fraction (%)	33.3 (12.3)	12.4	71.0	31.1 (11.2)	2.6	65.2
Multifidus	Total CSA (cm²)	3.36 (0.79)	1.58	5.62	2.92 (0.71)	1.74	4.72
	Lean CSA (cm²)	1.72 (0.59)	0.60	3.38	1.49 (0.50)	0.63	2.86
	Fat Fraction (%)	48.9 (9.7)	24.3	79.7	49.0 (9.6)	26.7	76.9
**L3**							
Erector spinae	Total CSA (cm²)	18.10 (3.83)	9.94	27.56	18.01 (4.13)	9.47	29.56
	Lean CSA (cm²)	11.47 (3.30)	3.60	20.18	11.41 (3.51)	4.50	22.73
	Fat Fraction (%)	37.1 (9.9)	20.0	78.2	37.2 (10.0)	19.7	68.2
Quadratus lumborum	Total CSA (cm²)	4.27 (1.63)	1.36	8.53	4.06 (1.60)	1.36	10.03
	Lean CSA (cm²)	3.12 (1.36)	0.91	6.64	2.93 (1.30)	0.73	7.68
	Fat Fraction (%)	27.9 (9.9)	14.4	61.4	28.6 (8.9)	13.8	62.4
Multifidus	Total CSA (cm²)	6.25 (1.63)	3.69	11.66	6.06 (1.62)	3.24	11.62
	Lean CSA (cm²)	3.38 (1.20)	1.15	6.90	3.23 (1.21)	1.27	7.87
	Fat Fraction (%)	45.9 (11.2)	22.7	77.8	46.8 (11.1)	21.3	72.3
**L5**							
Erector spinae	Total CSA (cm²)	7.65 (2.93)	2.44	16.00	7.60 (2.85)	1.94	16.00
	Lean CSA (cm²)	3.28 (1.61)	0.62	8.45	3.04 (1.54)	0.54	8.13
	Fat Fraction (%)	58.1 (11.8)	32.0	85.5	60.9 (11.3)	35.9	90.3
Quadratus lumborum	Total CSA (cm²)	2.82 (2.17)	0.01	7.47	3.30 (2.11)	0.01	6.54
	Lean CSA (cm²)	1.44 (1.24)	0.01	3.49	1.72 (1.08)	0.01	3.89
	Fat Fraction (%)	50.0 (19.4)	15.9	83.6	54.7 (18.6)	19.6	78.9
Multifidus	Total CSA (cm²)	10.33 (1.67)	4.13	14.00	10.30 (1.60)	5.52	15.72
	Lean CSA (cm²)	5.42 (1.53)	1.35	10.12	5.40 (1.44)	1.79	10.67
	Fat Fraction (%)	47.4 (12.2)	19.5	80.8	47.4 (11.4)	28.0	81.4
EMG parameters*							
Longissimus	Normalized MDF Decline (%)	14.8 (10.7)	−14.5	43.5	13.6 (10.3)	−8.4	47.3
	Initial MDF (Hz)	68.3 (12.4)	38.7	111.7	68.0 (14.0)	46.5	141.8
	Normalized MDF Slope (%/s)	−0.17 (0.16)	−0.86	0.27	−0.15 (0.13)	−0.46	0.48
Iliocostalis	Normalized MDF Decline (%)	12.5 (11.8)	−20.1	38.8	12.2 (10.9)	−16.7	42.7
	Initial MDF (Hz)	62.4 (12.8)	37.1	115.1	62.6 (14.5)	40.9	141.4
	Normalized MDF Slope (%/s)	−0.15 (0.13)	−0.63	0.13	−0.13 (0.15)	−0.50	0.67
Multifidus	Normalized MDF Decline (%)	14.7 (11.9)	−11.3	50.5	14.6 (10.5)	−9.8	39.8
	Initial MDF (Hz)	95.2 (19.6)	51.0	167.6	93.8 (18.0)	60.4	154.8
	Normalized MDF Slope (%/s)	−0.17 (0.14)	−0.61	0.20	−0.17 (0.12)	−0.49	0.07

⁎ EMG normalized median frequency decline and slope parameters: n = 94; All MRI parameters and EMG initial median frequency parameters: n = 105; SD, standard deviation; CSA, cross-sectional area; MDF, Median frequency.

### Bivariate associations

**Fig. 5:**
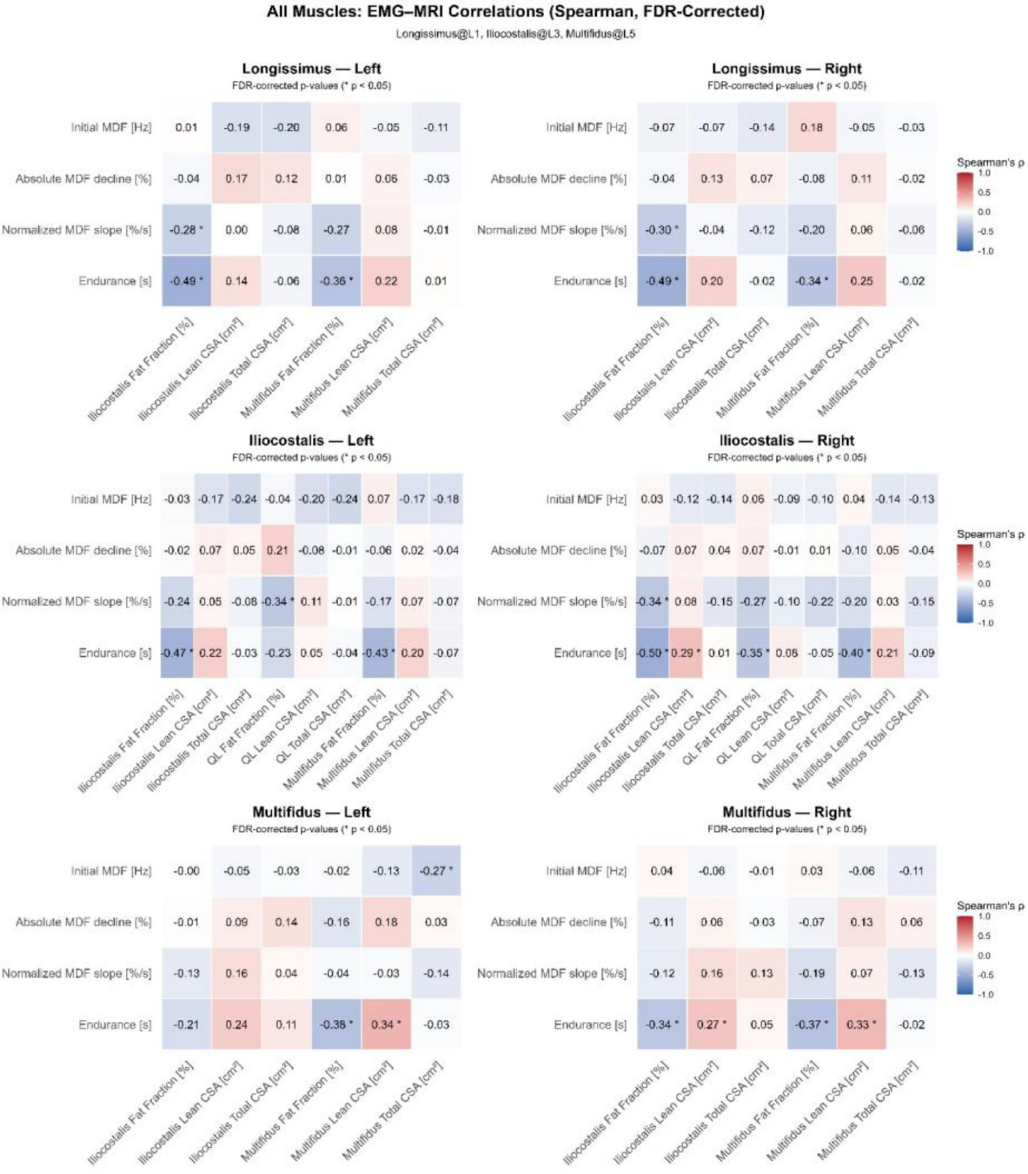
Heatmaps showing cross-correlations between EMG-derived fatigability, endurance and MRI-derived morphology. CSA: Cross-sectional area; MDF: Median frequency; FDR: False-discovery-rate; QL: Quadratus lumborum.

**Fig. 6:**
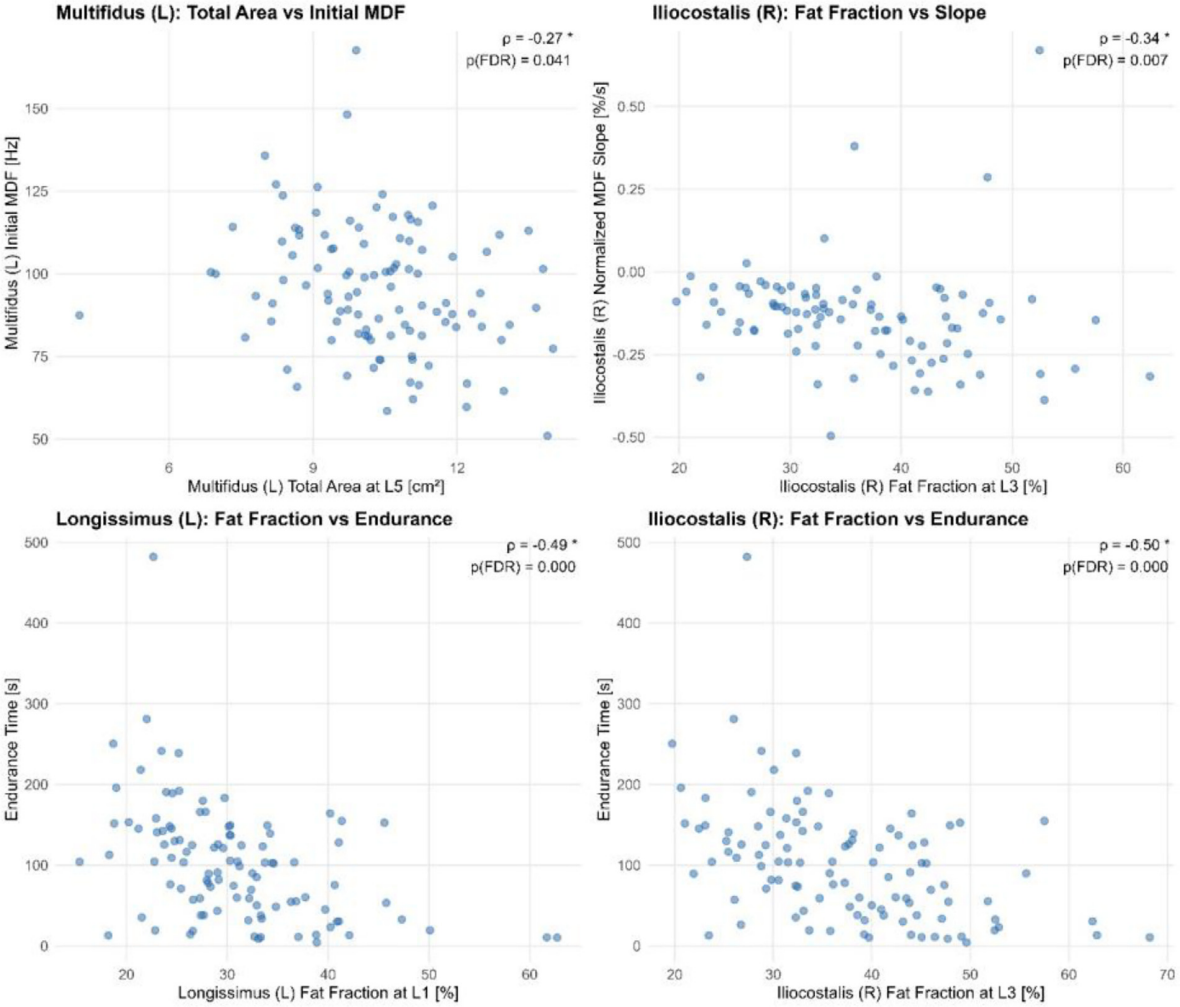
Scatterplots showing key associations between muscle morphology and functional parameters. Top left: Left multifidus total area vs. initial median frequency; top right: Right iliocostalis fat fraction vs. normalized median frequency slope; bottom left: Left longissimus fat fraction vs. endurance; bottom right: Right iliocostalis fat fraction vs. endurance. ρ: Spearman correlation coefficient; p(FDR): false-discovery-rate corrected p-value; n: sample size; MDF: median frequency; L: Left; R: Right. *: significant after false-discovery rate (FDR) correction.

Spearman correlation analysis (corrected for multiple comparisons using FDR) revealed consistent bilateral associations between fat fraction and endurance time across muscles and levels, with higher fat fraction associated with shorter endurance (−0.34 ≤ρ≤ −0.50; Fig. 5, Fig. 6). Endurance time correlated positively with lean, but not total muscle CSA, with significant associations for erector spinae at L3 and multifidus at L5 (0.298 ≤ρ≤ 0.34).

In contrast, associations between morphology and sEMG-derived fatigability parameters were side-specific and limited in scope. Normalized MDF slope correlated negatively with fat fraction at L1 and L3, but not at L5, with lateralization patterns varying by level (Fig. 5). Normalized MDF decline and initial MDF showed no significant associations with any morphological parameter after FDR correction. Overall, fat fraction-endurance associations were consistently bilateral, whereas relationships involving fatigability parameters demonstrated clear side-specificity.

### Multilevel models

**Fig. 7:**
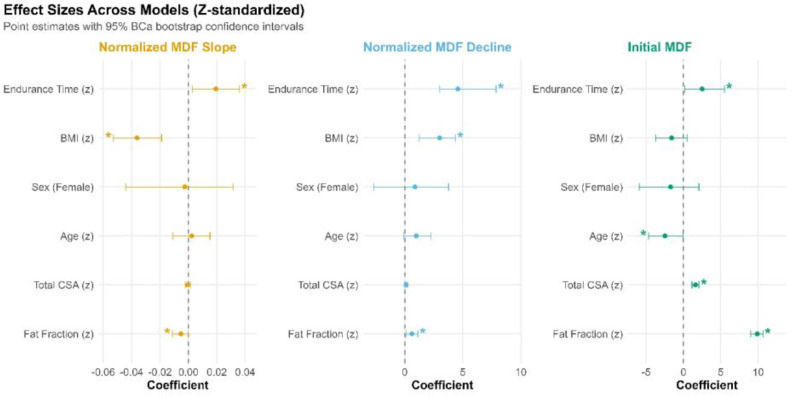
Standardized fixed-effect estimates from multilevel mixed-effects models predicting normalized MDF slope, normalized MDF decline, and initial MDF. Points show z-standardized coefficients with 95% BCa bootstrap confidence intervals. Dashed line indicates zero effect. BMI: body mass index; CSA: cross-sectional area; MDF: median frequency.

Multilevel models revealed that fat fraction was independently associated with all three fatigability outcomes: more negative normalized MDF slopes (estimate = −0.005, 95% CI [−0.011; −0.001]), greater normalized MDF decline (estimate = 0.619, 95% CI [0.15; 1.12]), and higher initial MDF (estimate = 9.883, 95% CI [9.01; 10.65]) (Table 3, Fig. 7). BMI was independently associated with normalized MDF slope (estimate = −0.036, 95% CI [−0.053; −0.019]) and normalized MDF decline (estimate = 3.008, 95% CI [1.23; 4.38]) but not with initial MDF. Endurance time was positively associated with normalized MDF slope (estimate = 0.019, 95% CI [0.003; 0.036]), normalized MDF decline (estimate = 4.577, 95% CI [3.00; 7.87]), and initial MDF (estimate = 2.501, 95% CI [0.19; 5.47]). Total muscle CSA was only associated with initial MDF (estimate = 1.599, 95% CI [1.14; 2.0]). Age was associated with initial MDF (estimate = −2.486, 95% CI [−4.70; −0.09]). Sex was no significant predictor in any model.

**Table 3:** Multilevel mixed linear models

Mixed models: Z-standardized predictors (all outcomes)															
	Normalized MDF slope					Normalized MDF decline					Initial MDF				
Predictors	Estimates	std. Error	Statistic	p	CI	Estimates	std. Error	Statistic	p	CI	Estimates	std. Error	Statistic	p	CI
(Intercept)	−0.155	0.015	−10.536	<.001	**[**−**0.174;** −0.132]*	13.096	1.292	10.137	<.001	[11.33; 14.77]*	74.290	1.978	37.552	<.001	[71.56; 77.02]*
Fat Fraction (z)	−0.005	0.003	−1.925	.054	**[**−**0.011;** −0.001]*	0.619	0.197	3.133	.002	[0.15; 1.12]*	9.883	0.495	19.949	<.001	[9.01; 10.65]*
Total CSA (z)	−0.001	0.002	−0.217	.828	[−0.002; 0.001]	0.119	0.171	0.696	.486	[−0.01; 0.26]	1.599	0.423	3.781	<.001	[1.14; 2.08]*
Age (z)	0.002	0.010	0.227	.821	[−0.011; 0.015]	1.000	0.879	1.137	.259	[−0.12; 2.25]	−2.486	1.305	−1.904	.060	**[**−**4.70;** −0.09]*
Sex (female)	−0.003	0.022	−0.113	.911	[−0.044; 0.031]	0.889	1.961	0.453	.652	[−2.68; 3.78]	−1.721	2.886	−0.596	.552	[−5.91; 2.08]
BMI (z)	−0.036	0.012	−2.993	.004	**[**−**0.053;** −0.019]*	3.008	1.059	2.840	.006	[1.23; 4.38]*	−1.592	1.560	−1.021	.310	[−3.74; 0.46]
Endurance (z)	0.019	0.011	1.731	.087	[0.003; 0.036]*	4.577	0.981	4.665	<.001	[3.00; 7.86]*	2.501	1.494	1.674	.097	[0.19; 5.47]*
Random effects															
ICC	0.65					0.71					0.42				
N	2 _side;_ 94 _ID_					2 _side_; 94 _ID_					105 _ID_				
Observations	1283					1283					1434				
Marginal R^2^ / Conditional R^2^	0.115 / 0.686					0.145 / 0.748					0.173 / 0.525				

MDF: Median frequency; CI: Confidence interval (bootstrapped, bias-adjusted); CSA: Cross-sectional area; BMI: Body mass index; ICC: Intra-class-correlation coefficient.

⁎ Statistically significant effect.

## Discussion

This study examined associations between paraspinal muscle morphology, endurance, and electromyographic fatigability in patients with sLSS scheduled for surgical decompression. The main finding is that paraspinal muscle fat fraction was consistently associated with both endurance capacity and sEMG-derived fatigability parameters, with higher fatty infiltration predicting shorter endurance times, a faster rate of MDF decline, and greater absolute MDF decrease during sustained trunk extension. Notably, muscle CSA showed limited associations with these outcomes, suggesting that muscle quality may be more relevant than muscle quantity for electromyographic fatigability characteristics in this population.

### Contextualization of morphology, endurance and fatigability parameters

The distribution of fatty infiltration across lumbar levels and muscles was consistent with prior observations that paraspinal muscle degeneration in sLSS is most pronounced at lower lumbar segments [8,45]. The observed magnitude of fatty infiltration was at the upper end of values reported in comparable cohorts of acute and chronic low back pain [9] and surgical LSS patients [5,8,[45], [46], [47], [48]]. In our cohort, fat fractions ranged from 30% (erector spinae, L1) to 61% (erector spinae, L5), with multifidus values around 50% across L1–L5, compared to approximately 20% to 40% typically reported in healthy populations [8,9,48]. These differences may partly stem from methodological variation, since most published studies estimated fat fraction using manual thresholding on T2‑weighted MRI, which can produce systematically different values than the VIBE Dixon–based approach used here.

Direct comparison of endurance values in our study with published normative values is difficult because of substantial protocol heterogeneity in trunk positioning, inclination angle, and whether tasks were performed to exhaustion or for a fixed duration. Compared to a recently published systematic review [49], endurance times in our study do not appear much lower than values reported for healthy elderly populations. However, a previous study [50] directly comparing a similar surgical LSS cohort to healthy elderly controls using a modified Biering-Sørensen test with an identical Roman chair setup (but the trunk in horizontal position) reported clearly shorter endurance times in surgical patients.

sEMG-derived fatigability parameters were lower than values reported in prior literature. Coorevits et al. [14] observed normalized MDF slopes of approximately −0.45%/s for multifidus, −0.40%/s for longissimus, and −0.20 to −0.25%/s for iliocostalis in young healthy controls performing the standard Biering-Sørensen test. Similarly, Mannion and Dolan [51] reported 33% to 36% reductions in MDF in young women. These differences may stem from the less demanding modified test protocol and the older, symptomatic study population. Furthermore, initial MDF values varied notably across muscles, with the multifidus demonstrating the highest baseline frequencies (∼94 Hz) compared to the longissimus (68 Hz) and iliocostalis (63 Hz). These inter-muscle differences may reflect underlying variation in fiber type composition and motor unit characteristics, discussed further in section (Fat fraction as a predictor of electromyographic fatigability).

### Side-specificity of morphology-fatigability associations

Correlation analyses demonstrated consistent bilateral associations between fat fraction and endurance, whereas several associations between fat fraction and sEMG-derived fatigability parameters were side-dependent. This discrepancy likely reflects the different nature of these outcomes: endurance is a global task-level measure influenced by bilateral trunk musculature, whereas sEMG fatigability metrics are sensitive to localized, side-specific factors.

The segmental and lateralized innervation of the paraspinal muscles, particularly the level-specific medial branch supply to the multifidus [52], makes these muscles susceptible to unilateral neural compromise. In this cohort, 55% of participants exhibited severe unilateral foraminal stenosis, and unilateral nerve root compression may preferentially alter ipsilateral muscle composition and activation. Additionally, sEMG is sensitive to side-specific differences in tissue composition, electrode placement, and signal quality, particularly in muscles with asymmetric fatty infiltration. The lateralized correlation patterns therefore likely reflect a combination of underlying unilateral pathology and methodological factors rather than measurement artifact alone.

### Fat fraction as a predictor of electromyographic fatigability

To interpret the multilevel model results it is necessary to consider the physiological basis of MDF change during sustained contractions. The change in sEMG MDF is mostly attributable to changes in muscle fiber conduction velocity [[16], [17], [18]], with fast-twitch Type IIA and Type IIX fibers contributing disproportionately due to their higher conduction velocities [53,54]. Other contributing factors include fatigue-related metabolic changes that impair sarcolemmal excitability [55] and shifts in motor unit recruitment [56]. MDF-derived fatigability indices should therefore be interpreted as composite markers reflecting the interaction of peripheral, metabolic, and neural contributions, rather than as a direct measure of any single mechanism. Importantly, although subcutaneous fat is a known spatial filter [57] that reduces sEMG amplitude and shifts power to lower frequencies, the fact that higher fat fractions were linked to higher (not lower) initial MDF values argues against a simple low‑pass filtering artifact and supports a physiological explanation.

A plausible explanation for the association between fat fraction and all three fatigability outcomes is a reduction in effective contractile reserve: as contractile fibers are replaced by adipose tissue, fewer functional motor units remain available to sustain force output. Consequently, the remaining units must be recruited earlier and driven at higher discharge rates, possibly accelerating the metabolic and ionic disturbances that manifest as conduction velocity slowing and spectral compression [[58], [59], [60]].

A second, more speculative explanation involves fiber-type remodeling. In a series of experimental studies, Mannion and colleagues demonstrated that the rate of MDF decline during sustained isometric back extension is influenced by fiber type distribution, with greater Type I fiber area predicting slower spectral decline [61], and fatigability of muscle fibers proceeding in the order Type IIX > Type IIA > Type I^61^. Histological studies in chronic low back pain have reported shifts toward Type IIX fibers at the expense of Type I, particularly with longer symptom duration and in surgical patients [36,62,63]. Such remodeling can occur without reductions in fiber size or total CSA [36], suggesting that electromyographic fatigability may be compromised through qualitative changes even without overt atrophy. Our data are consistent with this framework: an increased proportion of fast, fatigable fibers would elevate initial MDF and accelerate MDF decline during sustained contraction. However, this remains inferential, as our design did not include muscle biopsies, and evidence for systematic fiber-type alterations in degenerative spinal conditions remains conflicting [64,65].

Endurance time was positively associated with normalized MDF slope and, more strongly, with normalized MDF decline. The stronger association with normalized MDF decline is expected, as longer test durations inherently provide more time for cumulative spectral shifts to develop. Nevertheless, these results are generally in line with previous studies reporting associations between endurance and electromyographic fatigability metrics [14,51,66]. Importantly, fat fraction remained a significant predictor of both fatigability outcomes after accounting for endurance time, indicating that morphological degeneration contributes to electromyographic fatigability beyond what is explained by test duration alone.

BMI emerged as a strong and independent predictor of fatigability across models, most plausibly reflecting the greater absolute mechanical demands imposed by increased trunk mass during the Biering-Sørensen test. This effect was independent of paraspinal fat fraction, indicating that global body composition and local muscle quality represent distinct contributors to electromyographic fatigability. Age was also independently associated with lower initial MDF. A possible contributor is the commonly cited, although still debated, pattern of selective Type II fiber atrophy with advancing age [67].

### Strengths and limitations

Strengths of this study include the relatively large cohort combining quantitative MRI and sEMG assessments, Dixon-based MRI with machine-learning-assisted segmentation provided objective morphology measures, bilateral multi-muscle sEMG recordings enabled anatomically matched analyses, and multilevel statistical modeling accounted for the hierarchical data structure. Limitations include the cross-sectional design precluding causal inference, the modified Biering-Sørensen protocol limiting comparison with different protocols, and the absence of a healthy control group. sEMG limitations including crosstalk and signal attenuation by subcutaneous fat may have affected measurements. The exclusion of patients with endurance times below 15 seconds may have introduced selection bias. Finally, as a single-center study of surgical candidates, generalizability requires confirmation.

### Clinical implications and future directions

The present findings suggest that paraspinal fatty infiltration is associated with electromyographic fatigability metrics in sLSS. While morphological changes have not consistently been linked to pain severity in this population, they may have functional consequences relevant to daily activities. Patients with greater fatty infiltration demonstrated greater electromyographic fatigability and reduced endurance, which could contribute to the activity intolerance characteristic of neurogenic claudication.

Therefore, assessment of paraspinal muscle morphology may provide prognostically relevant information, as pronounced fatty infiltration indicates an increased risk of persistent functional limitations. Longitudinal studies including pre‑ and post‑decompression follow‑up and comparisons with healthy age‑matched controls will be important for distinguishing pathological changes from normal aging.

## Conclusions

In patients with sLSS awaiting surgical decompression, paraspinal muscle fat fraction was consistently associated with sEMG-derived fatigability parameters during sustained trunk extension. Higher fatty infiltration was associated with both higher rate and greater magnitude of MDF decline, independent of muscle size, endurance, age, and sex. These findings suggest that morphological changes in the paraspinal muscles of patients with sLSS have functional correlates that may contribute to activity limitations, with muscle quality appearing more relevant than muscle quantity for fatigability in this population.

## Author contributions

Conceptualization: DK, CNe, AM, CNü. Data curation: DK, GM, PS, AC. Formal Analysis: DK. Funding Acquisition: CNe, AM, SF, DI. Investigation: DK. Methodology: DK, CNü, PS, HJW. Project Administration: DK. Resources: CNe, AM. Software: DK, CNü, PS. Supervision: CNe, CNü, AM, SS, DI, SF, HJW. Visualization: DK. Writing - original draft: DK. Writing - review & editing: DK, CNü, CNe, SS, AM, GM, PS, HJW, AC, DI, SF.

## Ethical considerations

The study was approved by the regional ethics board (EKNZ Project ID 2022-01170).

## Consent to participate

All participants provided written informed consent prior to participating.

## Funding

This study was funded by the 10.13039/501100001711Swiss National Science Foundation (SNF #320030_204461).

## Data availability

The data underlying this manuscript will be provided by the authors upon reasonable request.

## Declaration of generative AI and AI-assisted technologies in the writing process

During the preparation of this work the authors used DeepL Write and ChatGPT to assist in enhancing the quality of the language. Claude Code and ChatGPT (R-Wizard) were used for assistance in code writing. After using these tools/services, the authors reviewed and edited the content as needed and take full responsibility for the content of the publication.

## Declaration of competing interest

The authors declared no potential conflicts of interest with respect to the research, authorship, and/or publication of this article.
